# Exacerbation Burden and Peripheral Airway Dysfunction as Determinants of Quality of Life in Bronchiectasis: Insights from Impulse Oscillometry

**DOI:** 10.3390/medicina62071381

**Published:** 2026-07-17

**Authors:** Vitaliano Nicola Quaranta, Giulia Amoroso, Alessio Marinelli, Maria Rosaria Vulpi, Andrea Portacci, Silvano Dragonieri, Marianna Cicchetti, Leonardo Boccassini, Rossella Novielli, Emanuela Resta, Giovanna Elisiana Carpagnano

**Affiliations:** 1 Institute of Respiratory Disease, Department of Basic Medical Sciences, Neuroscience and Sense Organs, University of Bari Aldo Moro, 70124 Bari, Italyg.amoroso4@phd.uniba.it (G.A.); alessio.marinelli@uniba.it (A.M.);; 2 Department of Medical and Surgical Sciences, University of Foggia, 71122 Foggia, Italy

**Keywords:** bronchiectasis, impulse oscillometry, quality of life, small airway dysfunction, exacerbation burden

## Abstract

*Background and Objectives*: Health-related quality of life (HRQoL) is a key outcome in bronchiectasis, reflecting the multidimensional burden of the disease. While exacerbation frequency is known to influence patient-reported outcomes, the contribution of peripheral airway dysfunction remains less clearly defined. Impulse oscillometry (IOS) provides a sensitive assessment of small airway resistance, but its relationship with HRQoL in bronchiectasis has not been fully explored. *Materials and Methods*: We conducted a cross-sectional observational study including 85 consecutive adults with non-cystic fibrosis bronchiectasis attending a second-level outpatient clinic. Clinical data, exacerbation history, radiological severity, lung function, inflammatory markers, and oscillometric parameters were collected. Peripheral airway resistance was assessed using the R5–R20 parameter derived from IOS. HRQoL was evaluated using the Quality of Life–Bronchiectasis (QoL-B) questionnaire. Associations between QoL-B score and clinical, functional, and inflammatory variables were explored using Spearman correlation and univariate linear regression. Variables with significant associations were entered into a multivariable linear regression model. *Results:* Patients had a median QoL-B score of 61.5 (IQR 55.5–80.0). Peripheral airway resistance (R5–20) was significantly associated with worse HRQoL in univariate analysis (β = −42.71; 95% CI −83.09 to −2.33; *p* = 0.038). Exacerbation frequency showed a strong inverse association with QoL-B (β = −5.74; 95% CI −8.60 to −2.89; *p* < 0.001). In multivariable analysis, exacerbations remained independently associated with poorer QoL (β = −5.365; *p* = 0.001), whereas the association with R5–20 was attenuated and no longer statistically significant. *Conclusions:* Peripheral airway dysfunction is associated with impaired HRQoL in bronchiectasis, but exacerbation burden appears to be the dominant independent determinant. These findings support the complementary role of oscillometry and clinical assessment in understanding patient-reported outcomes in bronchiectasis.

## 1. Introduction

Bronchiectasis is a chronic respiratory condition characterized by permanent bronchial dilatation, chronic inflammation, and recurrent exacerbations that significantly impair health-related quality of life (HRQoL) [1,2]. Although the disease affects approximately 500,000 individuals in the United States, with prevalence increasing substantially with age [1], recent European and international registry data have demonstrated that bronchiectasis represents a growing global health challenge, associated with substantial healthcare utilization, frequent hospitalizations, impaired quality of life, and considerable socioeconomic burden [2,3]. While the clinical burden of bronchiectasis is well established, the specific determinants of patient-reported outcomes remain incompletely understood, particularly regarding the relative contributions of physiological impairment versus symptom burden and exacerbation frequency.

Exacerbations represent critical events in the natural history of bronchiectasis, associated with progressive lung function decline, increased mortality, and substantial deterioration in quality of life [1,3]. The “frequent exacerbator phenotype” has emerged as a consistent and reproducible clinical pattern, with patients experiencing three or more exacerbations annually, demonstrating worse outcomes across multiple domains [3,4]. However, the relationship between exacerbation burden and HRQoL has not been systematically compared with other potential determinants, including peripheral airway dysfunction and inflammatory markers. Although bronchiectasis is classically characterized by neutrophilic airway inflammation, increasing evidence indicates that a subset of patients exhibits features of type 2 (T2) inflammation, including blood eosinophilia and elevated exhaled nitric oxide. These biomarkers have been associated with distinct clinical phenotypes and may influence symptom burden, treatment response, and disease severity. Therefore, Eosinophils (cells/µL) and FeNO were included as exploratory inflammatory variables to evaluate their potential relationship with health-related quality of life [1,4].

Peripheral airway dysfunction is increasingly recognized as a key pathophysiological feature of bronchiectasis, contributing to impaired mucociliary clearance, mucus retention, and susceptibility to infection [5,6,7]. Impulse oscillometry (IOS) provides a non-invasive method to assess small airway resistance and reactance, with parameters such as R5–20 (peripheral airway resistance), AX (reactance area), and FRES (resonant frequency) demonstrating correlations with disease severity, radiological extent, and bacterial colonization in bronchiectasis cohorts [5,6,7]. Despite these associations, the independent contribution of oscillometric parameters to patient-reported quality of life remains uncertain, particularly when considered alongside exacerbation history and other clinical variables.

The Quality of Life-Bronchiectasis (QoL-B) and Bronchiectasis Health Questionnaire (BHQ) are validated, disease-specific instruments that capture the multidimensional impact of bronchiectasis on patients’ daily functioning and well-being [8,9]. Previous studies have demonstrated that HRQoL correlates more strongly with subjective measures such as dyspnea and fatigue than with objective parameters including spirometry and radiological severity [10,11]. This suggests that patient-reported outcomes reflect unique aspects of disease burden not fully captured by conventional physiological assessments. However, the relative importance of peripheral airway dysfunction, as measured by oscillometry, compared to exacerbation frequency in determining HRQoL has not been adequately explored.

Understanding the independent predictors of health-related quality of life in bronchiectasis has important clinical implications. Identifying modifiable factors that most strongly influence patient-reported outcomes may improve patient stratification, optimize therapeutic strategies, and guide the selection of clinically meaningful endpoints for future interventional studies. Although exacerbation burden and peripheral airway dysfunction have each been associated with disease severity in bronchiectasis, their relative contribution to health-related quality of life has not been directly compared within the same multivariable analytical model. Therefore, it remains unclear whether physiological impairment assessed by impulse oscillometry provides clinically relevant information beyond that captured by exacerbation history and conventional lung function parameters [5,6,7,10,11].

To address this knowledge gap, the primary aim of this study was to identify the independent determinants of health-related quality of life in patients with bronchiectasis, with particular focus on the relative contributions of peripheral airway dysfunction (assessed by impulse oscillometry) and exacerbation frequency. Secondary aims included: (1) characterizing the clinical, radiological, and functional profile of a bronchiectasis cohort; (2) evaluating the associations between oscillometric parameters, inflammatory markers, and patient-reported outcomes; and (3) determining whether peripheral airway resistance remains independently associated with quality of life after adjustment for exacerbation burden.

## 2. Materials and Methods

### 2.1. Study Design and Setting

This was a cross-sectional, observational, monocentric study conducted at the Second-Level Bronchiectasis Outpatient Clinic of the Unit of Respiratory Diseases, Policlinico of Bari, Italy. Consecutive adult patients were enrolled between March 2024 and July 2025 after providing written informed consent. The study was conducted in accordance with the principles of the Declaration of Helsinki and local institutional regulations. The local Ethics Committee approved the study (Approval No. 159/DG, 7 February 2022).

### 2.2. Study Population

Adult patients (≥18 years) of both sexes with a confirmed diagnosis of non-cystic fibrosis bronchiectasis (NCFBE) were consecutively included. The diagnosis of bronchiectasis was established by high-resolution computed tomography (HRCT) of the chest. Patients with cystic fibrosis were excluded, as were those with traction bronchiectasis secondary to advanced fibrotic lung disease. Individuals unable to adequately cooperate with lung function testing or impulse oscillometry were also excluded. A total of 85 patients fulfilled the eligibility criteria and were included in the final analysis.

**Figure 1:**
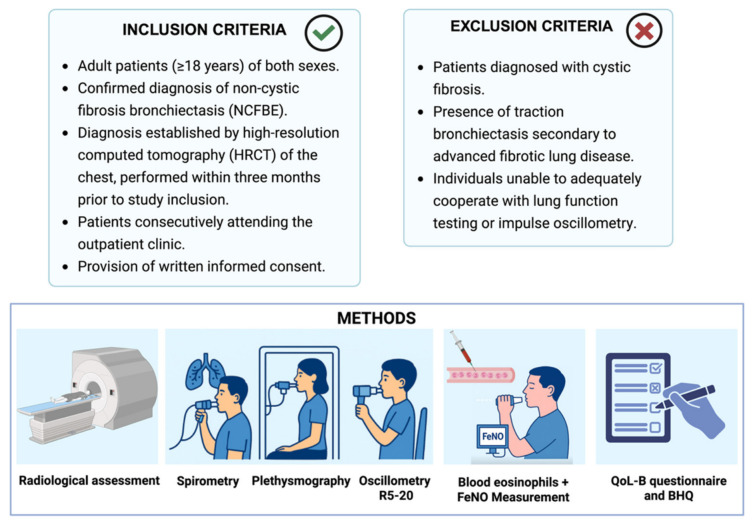
Study design, selection criteria, and multidimensional assessment protocol.

The study design, inclusion criteria, and multidimensional assessment protocol are summarized in Figure 1.

### 2.3. Radiological and Clinical Assessment

All patients underwent HRCT scanning within three months prior to study inclusion, with slice thickness ranging between 0.5 and 1.5 mm. Bronchiectasis was confirmed by an expert thoracic radiologist according to established criteria, including absence of normal bronchial tapering, visualization of bronchi within 1 cm of the costal pleura, and a broncho-arterial ratio greater than 1:1, typically presenting as the signet-ring sign [12]. Radiological extent was quantified using the Reiff score. Morphological patterns were categorized as tubular (cylindrical), varicose (ovalar), or saccular (cystic), the latter representing the most severe structural form [13].

Disease severity was assessed using validated multidimensional scoring systems. The Bronchiectasis Severity Index (BSI), which incorporates age, body mass index, percent predicted FEV_1_, hospitalizations in the previous two years, number of exacerbations in the previous year, mMRC dyspnea score, radiological extension, and chronic infection with *Pseudomonas aeruginosa* or other pathogens, was calculated for each patient. Scores range from 0 to 26 and classify disease as mild, moderate, or severe [14]. The FACED score, based on FEV_1_, age, chronic *Pseudomonas aeruginosa* infection, radiological extension, and dyspnea, was also determined. The number of exacerbations and hospitalizations occurring in the year preceding enrollment was recorded from clinical documentation.

### 2.4. Lung Function and Oscillometric Assessment

Pulmonary function testing was performed in accordance with international guidelines [15] using a Jaeger system (Essen, Germany). Spirometry and body plethysmography were carried out by experienced technicians under pneumologist supervision. Forced vital capacity (FVC), forced expiratory volume in one second (FEV_1_), residual volume (RV), and total lung capacity (TLC) were measured, along with diffusing capacity of the lung for carbon monoxide (DLCO) and the carbon monoxide transfer coefficient (KCO). The best of three reproducible maneuvers was selected, and values were expressed as percentages of predicted normal values [16].

Impulse oscillometry (IOS) was performed using the MasterScreen™ IOS system (Jaeger, Hoechberg, Germany) in accordance with the European Respiratory Society (ERS) recommendations for the forced oscillation technique [17]. Measurements were obtained during quiet tidal breathing for approximately 30 s. To minimize upper airway artifacts and ensure accurate assessment of respiratory impedance, the operator manually supported the patient’s cheeks throughout the maneuver.

At least three technically acceptable and reproducible measurements were recorded for each patient, as recommended by current ERS standards. The mean value of these maneuvers was calculated and used for subsequent analyses.

The oscillometric parameters evaluated included R5–R20, defined as the difference between resistance at 5 Hz and 20 Hz, which reflects peripheral airway resistance. Values greater than 0.07 kPa·s·L^−1^ were considered indicative of small airway dysfunction. Resonant frequency (Fres), defined as the frequency at which respiratory reactance equals zero, was recorded as an indicator of the balance between elastic and inertial forces within the respiratory system. Finally, AX, representing the area under the reactance curve between 5 Hz and Fres, was calculated as a marker of peripheral lung compliance and small airway involvement [18].

### 2.5. Inflammatory Markers

Although bronchiectasis is predominantly a neutrophilic disease, FeNO and peripheral blood eosinophil counts were included as exploratory biomarkers because previous studies have identified a subgroup of patients with evidence of T2 inflammation that may have distinct clinical characteristics and therapeutic implications [1,4]. Fractional exhaled nitric oxide (FeNO) was measured at flow rates of 50 mL/s and 350 mL/s using the FeNO+ analyzer (Medisoft-MGCD, Saint Paul, MN, USA), in accordance with manufacturer instructions and ERS recommendations [19]. Peripheral blood eosinophil counts were obtained from routine laboratory assessments performed at the time of clinical evaluation.

### 2.6. Patient-Reported Outcomes

Health-related quality of life was evaluated using the Quality of Life–Bronchiectasis (QoL-B) questionnaire [20] and the Bronchiectasis Health Questionnaire (BHQ) [9], both validated disease-specific instruments. The QoL-B total score was used as the primary dependent variable in regression analyses.

### 2.7. Collected Data

For each patient, comprehensive demographic, clinical, radiological, functional, inflammatory, therapeutic, and patient-reported variables were systematically collected, as detailed in Table 1 and Table 2.

Demographic data included age, sex, body mass index, smoking exposure expressed in pack-years, and smoking status categorized as never, former, or active smoker. Etiological classification of bronchiectasis was recorded, including idiopathic, obstructive-related, fibrotic sequelae, autoimmune, immunodeficiency-related, and CFTR-related forms, as well as the presence of cystic bronchiectasis.

Radiological and clinical severity parameters comprised the Reiff score, Bronchiectasis Severity Index, and documentation of exacerbations and hospitalizations in the previous year. Lung function variables included absolute and percentage predicted values of FEV_1_, FVC, RV, TLC, DLCO, and KCO. Oscillometric parameters included R5–20, AX, and FRES. Inflammatory markers consisted of FeNO measured at 50 mL/s and 350 mL/s and blood eosinophil count.

Comorbidities were systematically recorded and included cardiovascular diseases such as arterial hypertension, atrial fibrillation, myocardial infarction, stroke or transient ischemic attack, and congestive heart failure; metabolic and systemic conditions such as diabetes, chronic renal failure, liver cirrhosis, connective tissue diseases, rheumatoid arthritis, inflammatory bowel disease, and active neoplastic disease; respiratory comorbidities including COPD, asthma (including severe asthma), interstitial lung disease, ABPA, and chronic rhinosinusitis; as well as psychological conditions such as depression and anxiety and the presence of gastroesophageal reflux disease.

Current treatments were documented, including inhaled therapies such as LABA, LAMA, and inhaled corticosteroids; long-term macrolides or other antibiotic therapies; mucoactive agents including N-acetylcysteine; long-term oxygen therapy, non-invasive ventilation, and CPAP; as well as the regular performance of respiratory physiotherapy or physical activity at least once weekly.

Finally, patient-reported outcomes were collected using QoL-B total score and BHQ score.

**Figure 2:**
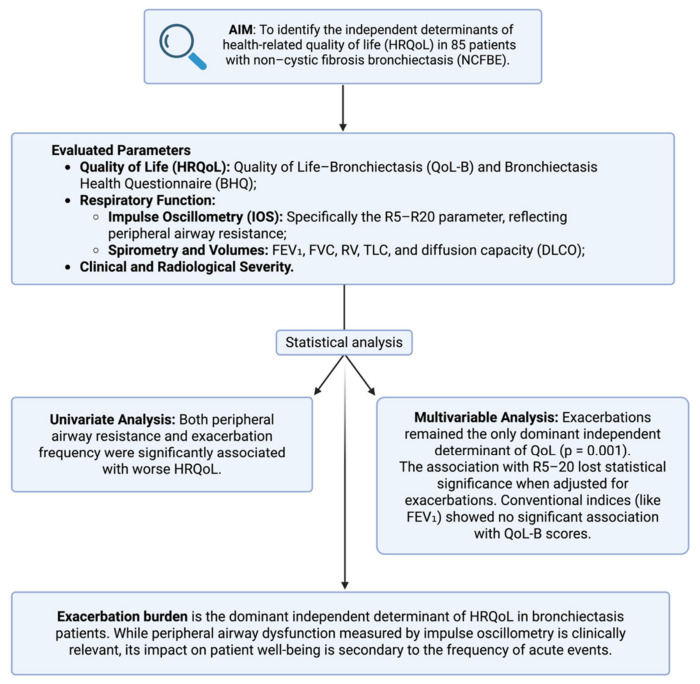
Analytical workflow and identification of determinants of HRQoL.

The analytical workflow is illustrated in Figure 2.

### 2.8. Statistical Analysis

Continuous variables were tested for normality using the Kolmogorov–Smirnov test and were expressed as median and interquartile range due to predominantly non-normal distribution. Categorical variables were presented as absolute frequencies and percentages.

Associations between QoL-B total score and demographic, clinical, radiological, functional, oscillometric, and inflammatory variables were explored using Spearman’s rank correlation coefficient. Univariate linear regression analyses were performed with QoL-B total score as the dependent variable. Variables reaching statistical significance in univariate analysis were subsequently entered into a multivariable linear regression model to identify independent determinants of health-related quality of life. Regression coefficients, 95% confidence intervals, and *p*-values were reported. A two-tailed *p*-value < 0.05 was considered statistically significant.

## 3. Results

### 3.1. Study Population and Demographic Characteristics

A total of 85 patients with bronchiectasis were included in the analysis. The cohort was predominantly male (76.5%) and older, with a median age of 67.5 years (IQR 60.5–70.5). Body mass index was generally within the normal range, with a median of 22.0 kg/m^2^ (IQR 20.0–26.5). Smoking exposure was modest overall, with a median of 10.0 pack-years (IQR 5.0–19.0). The majority of participants were never smokers (60.0%), while 36.5% were former smokers and only 3.5% were active smokers.

### 3.2. Etiology of Bronchiectasis

Idiopathic bronchiectasis represented the predominant etiology, accounting for 68.2% of cases. Secondary causes were less frequent and included fibrotic sequelae (9.4%), autoimmune diseases (9.4%), obstructive disease-related forms (7.1%), immunodeficiency (3.5%), and CFTR-related disease (2.4%). Cystic bronchiectasis was documented in 16.5% of patients, indicating that most individuals presented with non-cystic morphological patterns. The distribution of bronchiectasis etiologies is shown in Table 2.

### 3.3. Clinical and Radiologic Severity

Radiologic involvement was moderate in extent. The median Reiff score was 3.5 (IQR 2.5–4.5), suggesting limited lobar distribution in most patients. The Bronchiectasis Severity Index (BSI) showed a median value of 5.5 (IQR 4.0–8.0), consistent with an overall intermediate severity profile within the cohort.

Exacerbation burden emerged as a relevant clinical feature. In univariate regression analysis, the number of exacerbations in the previous year was strongly and inversely associated with health-related quality of life (QoL-B total score), with each additional exacerbation corresponding to a significant reduction in QoL (β = −5.74; 95% CI −8.60 to −2.89; *p* < 0.001). In contrast, hospitalizations in the previous year were not significantly associated with QoL.

### 3.4. Pulmonary Function

Spirometric evaluation revealed moderate airflow limitation. The median FEV_1_ was 1.84 L (IQR 1.58–2.55), corresponding to 75.0% of predicted values (IQR 65.0–95.5), while FVC was relatively preserved at 2.72 L (IQR 2.16–3.29), or 84.5% predicted (IQR 75.0–96.5). Lung volume analysis demonstrated evidence of air trapping, with residual volume reaching 128.5% predicted (IQR 97.0–149.5), whereas total lung capacity remained largely within normal limits (97.5% predicted, IQR 88.5–109.0). Diffusion capacity was moderately reduced, with DLCO at 69.0% predicted (IQR 58.5–77.5), while KCO was comparatively preserved at 92.0% predicted (IQR 72.0–100.5). Despite these physiological abnormalities, spirometric indices, lung volumes, and diffusion parameters were not significantly associated with QoL-B scores in univariate regression models.

### 3.5. Oscillometric Findings

Oscillometric assessment provided additional evidence of peripheral airway dysfunction. Median R5–20 was 0.095 kPa·s·L^−1^ (IQR 0.060–0.165), AX was 0.650 kPa·L^−1^ (IQR 0.295–1.540), and FRES was 15.3 Hz (IQR 13.8–20.95).

**Figure 3:**
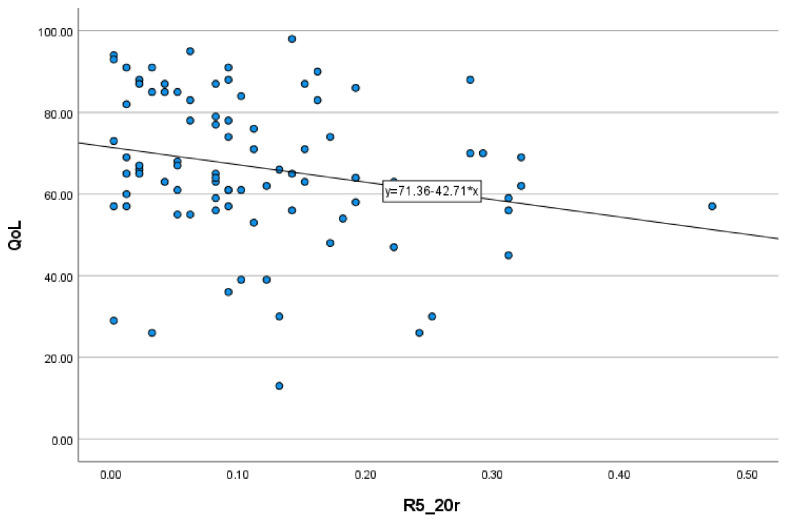
Relationship between peripheral airway resistance (R5–20) and health-related quality of life in patients with bronchiectasis.

Among oscillometric parameters, R5–20 showed a significant inverse association with QoL in univariate regression analysis (β = −42.71; 95% CI −83.09 to −2.33; *p* = 0.038), suggesting that greater small-airway dysfunction was linked to poorer patient-reported health status. The association between peripheral airway resistance (R5–20) and HRQoL is illustrated in Figure 3.

### 3.6. Inflammatory Markers

Inflammatory markers were generally low to moderate across the cohort. Median FeNO measured at 50 mL/s was 8.5 ppb (IQR 6.0–17.5), while FeNO at 350 mL/s was 5 [4,5,6,7,8,9,10]. The median blood eosinophil count was 195 cells/µL (IQR 80–385).

In univariate regression analysis, FeNO at 350 mL/s showed a modest positive association with QoL (β = 0.17; 95% CI −0.97 to 0.95; *p* = 0.109). FeNO at 50 mL/s and eosinophil counts were not significantly associated with QoL. In addition, peripheral blood neutrophil count, included as a marker of neutrophilic inflammation, was not significantly correlated with either QoL-B (Spearman’s ρ = 0.059, *p* = 0.597) or peripheral airway resistance (R5–20; Spearman’s ρ = −0.043, *p* = 0.703).

### 3.7. Patient-Reported Outcomes

Health-related quality of life was moderately impaired, with a median QoL-B total score of 61.5 (55.5–80.0). The Bronchiectasis Health Questionnaire (BHQ) score was 52.5 (47.0–55.0). BHQ demonstrated a strong positive association with QoL-B in univariate regression (β = 1.00; 95% CI 0.71–1.29; *p* < 0.001), supporting consistency between patient-reported outcome measures.

### 3.8. Multivariable Analysis

A multivariable linear regression model including R5–20 and exacerbations in the previous year was constructed to identify independent predictors of QoL. In this adjusted model, exacerbation frequency remained independently associated with lower QoL (β = −5.365; 95% CI −8.23 to −2.49; *p* = 0.001), whereas the association between R5–20 and QoL was attenuated and no longer statistically significant (*p* = 0.106). None of the other evaluated demographic, clinical, radiological, functional, or inflammatory variables demonstrated an independent association with HRQoL, as they did not reach statistical significance in the univariate analyses and were therefore not entered into the multivariable model (Table 3).

## 4. Discussion

This study demonstrates that peripheral airway dysfunction measured by R5–20 oscillometry and exacerbation frequency both contribute significantly to impaired health-related quality of life in patients with bronchiectasis, with exacerbations emerging as the dominant independent determinant in multivariable analysis. The significant univariate association between R5–20 and QoL underscores the clinical relevance of peripheral airway dysfunction, while the persistence of exacerbation frequency as an independent predictor reaffirms the central importance of exacerbation prevention. Importantly, none of the conventional spirometric indices, lung volumes, diffusion capacity parameters, inflammatory biomarkers, demographic characteristics, or radiological severity measures independently explained variability in HRQoL. These findings highlight the complementary roles of physiological assessment through oscillometry and clinical disease activity monitoring in understanding patient-reported outcomes, suggesting that comprehensive bronchiectasis management requires attention to both peripheral airway function and exacerbation burden.

R5–20, defined as the difference between resistance at 5 Hz and 20 Hz, is a key marker of small airway resistance and a hallmark of peripheral airway disease [5,6,7,8,9,9,10,11,12,13,14,15,16,17,18,19,20,21]. It has greater sensitivity than spirometry in detecting early dysfunction, often identifying abnormalities despite normal spirometric values [6,7,8,9,9,10,11,12,13,14,16,17,18,19,20,21]. In bronchiectasis, this reflects underlying mechanisms such as chronic inflammation, mucus plugging, and airway remodeling [6,7,8,9,9,10,11,12,13,14,15,16,17,18,19,20,21,22]. Consistently, IOS parameters—particularly R5–20—correlate with radiological and clinical severity [5,6,7]. In our study, the association between R5–20 and quality of life suggests that peripheral airway dysfunction contributes to impaired patient-reported outcomes, likely through its impact on dyspnea, exercise limitation, and mucus clearance [10,21].

A key strength of oscillometry is its ability to detect peripheral airway abnormalities with greater sensitivity than spirometry, particularly in mild-to-moderate disease [7,15]. In the present cohort, despite median FEV_1_ of 75.0% predicted indicating only moderate airflow limitation, oscillometric parameters revealed substantial peripheral airway dysfunction. This dissociation between spirometry and oscillometry has been consistently observed: in patients with HRCT scores ≤5, abnormal IOS parameters were more common than reduced FEV_1_, with increased frequency dependence serving as a sensitive marker of mild bronchiectasis [6]. Oscillometry offers several practical advantages that enhance its clinical utility [15]. The ability of oscillometry to differentiate bronchiectasis from other obstructive lung diseases represents an additional clinical application: in a comparative study, expiratory phase measurements revealed significantly higher airway resistance in COPD compared to bronchiectasis patients with similar FEV_1_ values, suggesting disease-specific patterns of airway dysfunction [23]. This discriminatory capacity may aid in phenotyping patients with overlapping clinical features and guide targeted therapeutic interventions.

The attenuation of the R5–20 association with QoL in multivariable analysis (*p* = 0.106) when adjusted for exacerbation frequency suggests a complex interrelationship between peripheral airway dysfunction and exacerbation risk. This finding does not diminish the clinical importance of R5–20; rather, it indicates that peripheral airway dysfunction may influence QoL primarily through its contribution to exacerbation susceptibility. The pathophysiological link is well-established: small airway obstruction impairs mucociliary clearance, promotes mucus retention, and creates an environment conducive to bacterial colonization and recurrent infections [6,22]. Evidence supporting this mechanistic pathway includes the observation that higher IOS parameters are associated with *Pseudomonas aeruginosa* infection and greater bacterial burden, both of which predict increased exacerbation frequency [6]. Interventions targeting peripheral airway dysfunction, such as oscillatory positive expiratory pressure devices, have shown improvements in R5–20 and secretion clearance, suggesting a potential role in reducing exacerbation risk [24]. IOS parameters remain stable during exacerbations, reflecting chronic structural abnormalities rather than acute changes [6]. This contrasts with spirometry and symptoms, which fluctuate with disease activity, indicating that R5–20 may represent a trait marker, whereas exacerbations reflect a dynamic state. Ultimately, both influence QoL, but exacerbations have a more immediate impact on patient well-being [1,3].

**Figure 4:**
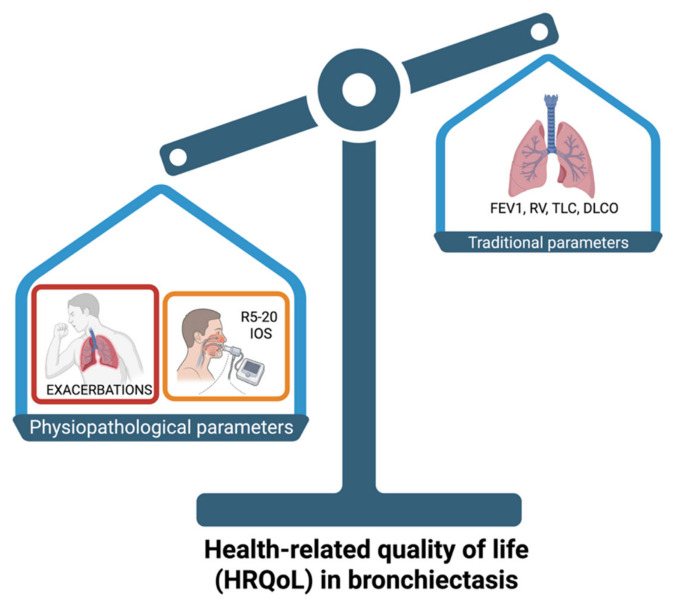
Conceptual framework of determinants of health-related quality of life in bronchiectasis.

Exacerbation frequency remained an independent predictor of QoL, reinforcing the central role of exacerbation prevention in bronchiectasis management [1,3,4,4,5,6,7,8,9,9,10,11,12,13,14,15,16,17,18,19,20,21,22,23,24]. Exacerbations are clinically impactful events associated with symptom worsening and cumulative adverse outcomes [25,26]. Evidence from the EMBARC study confirms that frequent exacerbations predict poorer QoL and increased mortality [3]. In our cohort, exacerbations showed a strong negative association with QoL, supporting their major impact on patient well-being. Symptom severity and exacerbations are closely interrelated, suggesting that combined strategies targeting both may improve outcomes [26]. Accordingly, guideline-recommended therapies such as long-term macrolides or inhaled antibiotics can reduce exacerbations and improve QoL [1,4,27]. Although bronchiectasis is classically considered a neutrophilic inflammatory disease, we expanded our inflammatory analysis by evaluating peripheral blood neutrophil count as a marker of systemic neutrophilic inflammation [1,4]. In our cohort, neutrophil count was not significantly associated with either peripheral airway dysfunction (R5–20) or HRQoL, suggesting that circulating neutrophils do not reflect the degree of small airway impairment or patient-reported disease burden. We also evaluated FeNO and Eosinophils (cells/µL) as exploratory biomarkers because accumulating evidence indicates that a subset of patients exhibits features of type 2 (T2) inflammation. However, neither Eosinophils (cells/µL) nor FeNO 50 showed an independent association with HRQoL. Collectively, these findings suggest that circulating inflammatory biomarkers, whether reflecting neutrophilic or T2 inflammatory pathways, contributed less to HRQoL than clinical factors such as exacerbation burden. The present findings support the integration of oscillometry into comprehensive bronchiectasis assessment, complementing rather than replacing spirometry and other conventional measures [7]. Integrating these findings, Figure 4 proposes a conceptual framework summarizing the relationships between exacerbation burden, peripheral airway dysfunction, and health-related quality of life in bronchiectasis. The model highlights exacerbations as the dominant independent determinant, while small airway dysfunction contributes as a complementary physiological factor influencing patient-reported outcomes.

Several clinical applications emerge from these findings. R5–20 may identify patients with peripheral airway dysfunction despite preserved spirometry, aiding risk stratification and therapeutic decisions, including bronchodilator use [5,6,7]. Serial oscillometry may detect early functional decline and enable longitudinal monitoring, given the stability of IOS parameters during exacerbations [6,7,8,9,9,10,11,12,13,14,15,16,17,18,19,20,21]. Oscillometry is also responsive to airway clearance interventions, supporting its role in treatment evaluation [24]. Patients with elevated R5–20 may represent a distinct small airway phenotype, potentially benefiting from targeted therapies [4,5,27]. In a study correlating functional and morphological indices, R5 showed the strongest association with normalized bronchial wall thickness (Pi10; r = 0.57), while both the Bronchiectasis Severity Index (BSI) and dyspnea scores correlated more closely with R5–20 than with spirometric parameters, further supporting the clinical relevance of oscillometry in assessing disease severity and symptom burden [28].

The absence of significant associations between spirometric parameters, lung volumes, and QoL in this cohort aligns with extensive literature demonstrating weak-to-moderate correlations between objective physiological measures and patient-reported outcomes [10,11]. In a systematic review of 43 studies, HRQoL showed stronger correlations with subjective measures such as dyspnea (r = 0.55) compared to FEV_1_% predicted (r = −0.31) [11]. The moderate air trapping (RV 128.5% predicted) and reduced diffusion capacity (DLCO 69.0% predicted) observed in this cohort likely contribute to dyspnea and exercise limitation, but their impact on QoL appears mediated through symptom experience rather than as direct independent determinants. The strong correlation between QoL-B and BHQ scores (*p* < 0.001) validates the consistency of patient-reported outcomes and supports the use of these disease-specific instruments in clinical practice and research [8,9].

This study has several limitations. The cross-sectional design precludes causal inference regarding the relationships between R5–20, exacerbations, and QoL. Longitudinal studies are needed to determine whether baseline R5–20 predicts future exacerbation risk and QoL trajectories, and whether changes in R5–20 following therapeutic interventions correlate with clinical improvements. The predominantly idiopathic etiology (68.2%) and mild-to-moderate severity profile (median BSI 5.5) may limit generalizability to more severe or etiologically diverse populations. The modest sample size (*n* = 85) may have limited statistical power to detect smaller independent effects of R5–20 in multivariable models, particularly given the strong association with exacerbations. Future research should focus on several key areas: prospective studies examining whether R5–20 predicts future exacerbation risk independently of prior exacerbation history would clarify its prognostic value and potential role in risk stratification; intervention studies assessing whether therapies that improve R5–20 translate into clinically meaningful benefits in terms of exacerbation reduction and improvement in health-related quality of life are also warranted. In this context, integrating oscillometric parameters into longitudinal monitoring strategies may provide a more comprehensive assessment of disease trajectory, complementing traditional clinical and spirometric indices.

Furthermore, the potential role of oscillometry in identifying distinct phenotypes of bronchiectasis deserves further investigation. Patients characterized by predominant peripheral airway dysfunction may represent a specific subgroup with unique pathophysiological mechanisms and therapeutic needs, potentially benefiting from targeted interventions aimed at improving small airway function and mucus clearance. The incorporation of oscillometric markers into multidimensional severity scores or treatable traits frameworks may enhance personalized management strategies in bronchiectasis.

From a clinical perspective, these findings reinforce the central importance of exacerbation prevention as a primary therapeutic goal, while also highlighting the added value of assessing peripheral airway dysfunction to better characterize disease burden. A combined approach integrating clinical history, patient-reported outcomes, and advanced physiological assessment may therefore provide the most accurate representation of disease impact and guide more effective, individualized treatment strategies.

In conclusion, peripheral airway dysfunction is significantly associated with impaired health-related quality of life in bronchiectasis, although its effect appears to be partially mediated by exacerbation burden. Exacerbation frequency remains the dominant independent determinant of patient-reported outcomes, underscoring the importance of strategies aimed at reducing exacerbations. These findings support the complementary role of impulse oscillometry and clinical assessment in the multidimensional evaluation of bronchiectasis and highlight the need for integrated approaches to optimize patient-centered care.

## Figures and Tables

**Table 1:** Baseline demographic characteristics, comorbidities, and treatments of the study population (*N* = 85).

Parameter (*N* = 85)	Median [IQR] or *n* (%)
**Demographics** **and Anthropometrics**	
Age (years)	67.5 [60.5–70.5]
Male sex	65 (76.5%)
Female sex	20 (23.5%)
BMI (kg/m^2^)	22.0 [20.0–26.5]
Smoking exposure (pack-years)	10.0 [5.0–19.0]
Never smoker	51 (60.0%)
Former smoker	31 (36.5%)
Active smoker	3 (3.5%)
**Comorbidities**	
Arterial hypertension	28 (32.9%)
Atrial fibrillation	7 (8.2%)
Myocardial infarction	2 (2.4%)
Stroke/TIA	3 (3.5%)
Congestive heart failure	5 (5.9%)
Chronic renal failure	5 (5.9%)
Liver cirrhosis	1 (1.2%)
Diabetes	8 (9.4%)
COPD	8 (9.4%)
Asthma	14 (16.5%)
Severe asthma	6 (7.1%)
Chronic rhinosinusitis	31 (36.5%)
Interstitial lung disease	2 (2.4%)
ABPA	2 (2.4%)
Connective tissue disease	10 (11.8%)
Rheumatoid arthritis	1 (1.2%)
Inflammatory bowel disease	4 (4.7%)
Depression	9 (10.6%)
Anxiety	9 (10.6%)
Active neoplastic disease	3 (3.5%)
GERD	46 (54.1%)
**Treatment**	
LABA	33 (38.8%)
LAMA	25 (29.4%)
Inhaled corticosteroids	29 (34.1%)
Long-term macrolides	3 (3.5%)
Long-term oral antibiotics	0 (0%)
Long-term inhaled antibiotics	2 (2.4%)
Long-term N-acetylcysteine	38 (44.7%)
Other mucoactive treatment	7 (8.2%)
Long-term oxygen therapy	3 (3.5%)
Long-term NIV	0 (0%)
Long-term CPAP	2 (2.4%)
Respiratory physiotherapy ≥1/week	35 (41.2%)
Physical activity ≥1/week	17 (20.0%)

Continuous variables are presented as median [interquartile range (IQR)], whereas categorical variables are presented as number (percentage). **Abbreviations**: BMI, body mass index; TIA, transient ischemic attack; COPD, chronic obstructive pulmonary disease; ABPA, allergic bronchopulmonary aspergillosis; GERD, gastroesophageal reflux disease; LABA, long-acting β_2_-agonist; LAMA, long-acting muscarinic antagonist; NIV, non-invasive ventilation; CPAP, continuous positive airway pressure.

**Table 2:** Disease characteristics, lung function, oscillometric parameters, inflammatory markers, and patient-reported outcomes of the study population (*N* = 85).

Parameter (*N* = 85)	Median [IQR] or *n* (%)
**Etiology**	
Obstructive-related etiology	6 (7.1%)
CFTR-related etiology	2 (2.4%)
Fibrotic sequelae	8 (9.4%)
Immunodeficiency (acquired/genetic)	3 (3.5%)
Autoimmune etiology	8 (9.4%)
Cystic bronchiectasis	14 (16.5%)
**Radiologic and Clinical Severity**	
Reiff score	3.5 [2.5–4.5]
Bronchiectasis Severity Index	5.5 [4.0–8.0]
**Lung Function**	
FEV_1_ (L)	1.84 [1.58–2.55]
FEV_1_ (% predicted)	75.0 [65.0–95.5]
FVC (L)	2.72 [2.16–3.29]
FVC (% predicted)	84.5 [75.0–96.5]
RV (L)	2.57 [2.20–3.51]
RV (% predicted)	128.5 [97.0–149.5]
TLC (L)	5.44 [4.87–6.57]
TLC (% predicted)	97.5 [88.5–109.0]
DLCO (% predicted)	69.0 [58.5–77.5]
KCO (% predicted)	92.0 [72.0–100.5]
**Oscillometry**	
R5–20 (kPa·s·L^−1^)	0.095 [0.060–0.165]
AX (kPa·L^−1^)	0.650 [0.295–1.540]
FRES (Hz)	15.3 [13.80–20.95]
**Inflammatory Markers**	
FeNO 50 (ppb)	8.5 [6.0–17.5]
FeNO 350 (ppb)	5 [4–10]
Eosinophils (cells/µL)	195 [80–385]
Blood neutrophils (cells/µL)	2850 [2200–3525]
**Patient-Reported Outcomes**	
QoL-B total score	61.5 [55.5–80.0]
BHQ	52.5 [47.0–55.0]

Continuous variables are presented as median [interquartile range (IQR)], whereas categorical variables are presented as number (percentage). **Abbreviations**: CFTR, cystic fibrosis transmembrane conductance regulator; FEV_1_, forced expiratory volume in one second; FVC, forced vital capacity; RV, residual volume; TLC, total lung capacity; DLCO, diffusing capacity of the lung for carbon monoxide; KCO, carbon monoxide transfer coefficient; R5–20, difference between resistance at 5 Hz and 20 Hz; AX, reactance area; FRES, resonant frequency; FeNO, fractional exhaled nitric oxide; QoL-B, Quality of Life–Bronchiectasis questionnaire; BHQ, Bronchiectasis Health Questionnaire.

**Table 3:** Linear Regression Dependent Variable: QoL-B questionnaire.

	Linear Univariate Regression		Multivariate Regression	
Parameter	Value (95% CI)	*p* Value		
**Oscillometry**				
R5–20 (kPa·s·L^−1^)	−42.71 (−83.09; −2.33)	0.038 *	−31.264 (−69.34; 8.81)	0.106
**Clinical Burden/Severity**				
Exacerbations (previous year)	−5.74 (−8.60; −2.89)	<0.001 *	−5.365 (−8.23; −2.49)	0.001
Hospitalisations (previous year)	−1.72 (−12.38; 8.94)	0.749		
BSI score	−0.61 (−1.70; 0.49)	0.274		
Reiff score	−0.34 (−1.75; 1.07)	0.631		
BHQ score	1.00 (0.71; 1.29)	<0.001		
**Spirometry**				
FEV_1_ (L)	2.87 (−2.49; 8.23)	0.289		
FEV_1_ (% predicted)	0.00 (−0.19; 0.19)	0.997		
FVC (L)	2.73 (−1.86; 7.32)	0.240		
FVC (% predicted)	−0.01 (−0.22; 0.21)	0.952		
**Lung Volumes**				
RV (L)	1.87 (−1.73; 5.46)	0.304		
RV (% predicted)	0.03 (−0.04; 0.11)	0.374		
TLC (L)	1.78 (−1.22; 4.79)	0.241		
TLC (% predicted)	0.09 (−0.11; 0.29)	0.379		
**Diffusion Capacity**				
DLCO	0.01 (−0.01; 0.02)	0.454		
DLCO (% predicted)	0.14 (−0.11; 0.39)	0.254		
KCO_abs	0.01 (−0.04; 0.06)	0.645		
KCO (% predicted)	0.11 (−0.14; 0.35)	0.387		
**Inflammatory Markers**				
FeNO 50 (ppb)	0.15 (−0.36; 0.65)	0.560		
FeNO 350 (ppb)	0.17 (−0.97; 0.952)	0.109		
Eosinophils (cells/µL)	−0.00 (−0.03; 0.03)	0.943		
Blood neutrophils (cells/µL)	0.001 (−0.003–0.005)	0.750		
**Demographics/Anthropometrics**				
Age (years)	−0.01 (−0.29; 0.28)	0.967		
BMI (kg/m^2^)	−0.65 (−1.60; 0.29)	0.173		
Pack-years	−0.05 (−0.43; 0.32)	0.771		

Values are reported as β coefficients (95% confidence intervals [CI]). Only variables with a statistically significant association in the univariate analysis were entered into the multivariable regression model. Statistical significance was defined as *p* < 0.05. **Abbreviations:** QoL-B, Quality of Life–Bronchiectasis questionnaire; CI, confidence interval; R5–20, difference between respiratory resistance at 5 Hz and 20 Hz; BSI, Bronchiectasis Severity Index; BHQ, Bronchiectasis Health Questionnaire; FEV_1_, forced expiratory volume in one second; FVC, forced vital capacity; RV, residual volume; TLC, total lung capacity; DLCO, diffusing capacity of the lung for carbon monoxide; KCO, carbon monoxide transfer coefficient; FeNO, fractional exhaled nitric oxide; BMI, body mass index. * *p* < 0.05.

## Data Availability

The data presented in this study are available from the corresponding author upon reasonable request. The data are not publicly available due to privacy and ethical restrictions related to patient information.

## Abbreviations

BMI = Body Mass Index; CFTR = Cystic Fibrosis Transmembrane Conductance Regulator; NTM = Non-tuberculous mycobacteria; COPD = Chronic Obstructive Pulmonary Disease; GERD = Gastroesophageal Reflux Disease; ABPA = Allergic Bronchopulmonary Aspergillosis; FEV_1_ = Forced Expiratory Volume in 1 s; FVC = Forced Vital Capacity; RV = Residual Volume; TLC = Total Lung Capacity; DLCO = Diffusing Capacity of the Lung for Carbon Monoxide; KCO = Carbon Monoxide Transfer Coefficient; FeNO = Fractional exhaled Nitric Oxide; BHQ = Bronchiectasis Health Questionnaire; LABA = Long-Acting Beta_2_-Agonist; LAMA = Long-Acting Muscarinic Antagonist; ICS = Inhaled Corticosteroid; LTOT = Long-Term Oxygen Therapy; NCFBE = Non-Cystic Fibrosis Bronchiectasis; HRCT = High-Resolution Computed Tomography; IOS = Impulse Oscillometry; QoL-B = Quality of Life–Bronchiectasis.
